# Research on the Crushing Process of PELE Casing Material Based on the Crack-Softening Algorithm and Stochastic Failure Algorithm

**DOI:** 10.3390/ma11091561

**Published:** 2018-08-30

**Authors:** Liangliang Ding, Jingyuan Zhou, Wenhui Tang, Xianwen Ran, Ye Cheng

**Affiliations:** 1 College of Liberal Arts and Sciences, National University of Defense Technology, Changsha 410073, China; dingliangliang14@nudt.edu.cn (L.D.); zhoujingyuan12@163.com (J.Z.); ranxianwen@nudt.edu.cn (X.R.); 2 School of Basic Sciences for Aviation, Naval Aviation University, Yantai 264001, China; chengye2014@163.com

**Keywords:** PELE, Penetration with Enhanced Lateral Efficiency, crack softening algorithm, stochastic failure algorithm, AUTODYN

## Abstract

In order to more realistically reflect the penetrating and crushing process of a PELE (Penetration with Enhanced Lateral Efficiency) projectile, the stochastic failure algorithm and crack-softening algorithm were added to the corresponding material in this paper. According to the theoretical analysis of the two algorithms, the material failure parameters (stochastic constant *γ*, fracture energy *G*_f_, and tensile strength *σ*_T_) were determined. Then, four sets of simulation conditions ((a) no crack softening, (b) no stochastic failure, (c) no crack softening and no stochastic failure, and (d) crack softening and stochastic failure) were designed to qualitatively describe the influences of the failure algorithms, which were simulated by the finite element analysis software AUTODYN. The qualitative comparison results indicate that the simulation results after adding the two algorithms were closer to the actual situation. Finally, ten groups of simulation conditions were designed to quantitatively analyze the coincidence degree between the simulation results and the experimental results by means of two parameters: the residual velocity of the projectile and the maximum radial velocity of fragments. The results show that the simulation results coincide well with the experimental results and the errors were small. Therefore, the ideas proposed in this paper are scientific, and the conclusions obtained can provide guidance for engineering research.

## 1. Introduction

**Figure 1:**
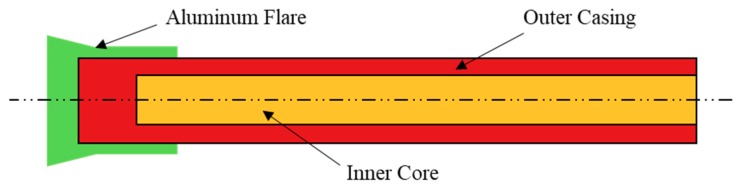
Longitudinal section of PELE projectile.

**Figure 2:**
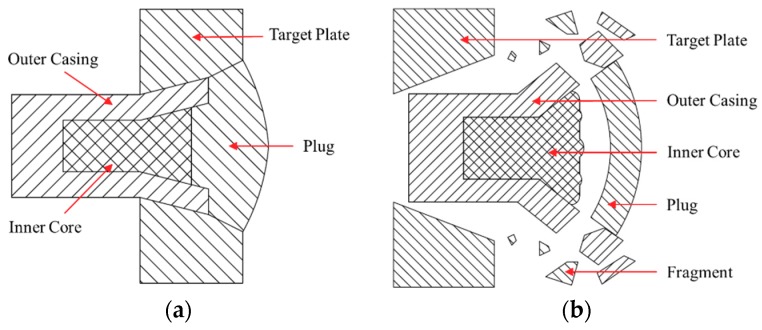
Different penetration state of a PELE projectile: (**a**) initial penetration state; (**b**) completely perforating state.

The PELE (Penetration with Enhanced Lateral Efficiency) projectile [1,2] is a new type of armor-piercing warhead that can transform part of the axial kinetic energy into radial kinetic energy by using the differences in material properties between the projectile casing and the inner core. The PELE projectile was originally developed jointly by the French–German Research Institute Saint Louis (ISL), the Diehl Munitionssysteme, and the GEKE Technology [1,2,3,4,5]. This new type of projectile is mainly composed of the outer casing and an inner core, and its structural diagram is shown in Figure 1. The outer casing is generally made of a dense metal, such as tungsten alloy and steel, and the inner core is generally made of a material with a relatively low density such as aluminum and plastic. Compared with the conventional armor-piercing projectiles, a PELE projectile can form a larger transverse reaming during penetration and produce fragment effects after perforating the target plate (as shown in Figure 2), which makes it significantly better than the conventional armor-piercing projectiles, especially in terms of damage efficiency.

In order to investigate the damage efficiency and the influencing factors of PELE projectiles, a lot of research has been carried out from the following three aspects: experiment, theoretical analysis, and numerical simulation. Rheinmetall GmbH [3] conducted a series of tests of large-caliber PELE projectiles on reinforced concrete targets, brick walls, and sandbag walls, and the reaming of target plates under different conditions were measured. Paulus et al. [5] reported a large number of experimental results of the PELE projectile impacting the target plate, which were realized with the powder gun and the light gas gun. In the experiments, the axial residual velocity of the projectile and the radial velocity of fragments were measured by using flash X-ray photography. Paulus’ test results are the most representative at present, and this paper will also take his test results as a reference for comparison. Zhu et al. [6,7] used the ø12.7 mm PELE to impact a 2 mm thick armored steel target plate, and the post-effect target was a 1 mm thick aluminum plate. The effective dispersion area of fragments on the post-effect targets and the number of fragments were measured. Jiang et al. [8] used the ø30 mm PELE to impact the spaced target plates, which were composed of three 3 mm thick A3 steel and a 15 mm thick armored steel. Tu et al. [9] carried out experiments on the impact of a ø30 mm PELE on different thickness target plates, where the dispersion of fragments and the residual velocity of projectiles were measured. To sum up, the theoretical analysis model of a PELE projectile usually includes two parts: the axial penetration of the projectile and the fragment radial dispersion. The axial penetration process of a projectile is basically described by the plug model, and there is little difference between the various theoretical models. Therefore, many scholars mostly focused on the fragment dispersion models. Paulus [5], Zhu [6], Du [10], and Fan [11] simplified the crushing process of a PELE projectile, and the calculation methods of the maximum radial velocity of fragments were put forward. Based on Mott fragmentation theory, Verreault et al. [12] proposed a model to describe the axial crushing of a PELE projectile and the fragment dispersion. Although the experiment can give the true crushing state of PELE casing material, the test cost and period are longer, and the intermediate crushing process of PELE casing material cannot be given. Therefore, many scholars have studied the fragment effects of PELE casing material by numerical simulation. Paulus et al. [2,5] used LS-DYNA-3D code to simulate the crushing process of PELE casing material under different working conditions and compared it with the theoretical model. Wang et al. [13] used the three-dimensional SPH (Smoothed Particle Hydrodynamics) dynamics algorithm to simulate the fragment effects of PELE casing material at a normal projectile velocity, and the effects of target thickness, target material, and projectile velocity on the projectile crushing were compared. Verreault [14] carried out numerical simulations by using the AUTODYN software to verify his proposed model for describing the fragment effects of PELE casing material.

This paper aims to study the crushing process of PELE casing material from the aspect of numerical simulations. As seen from the extensive literature research, most existing numerical simulations usually adopt the relatively simple failure model to characterize the penetration and crushing process of PELE casing material, which leads to many problems such as it cannot reflect the random failure of materials. Therefore, this paper hopes to make the numerical simulation results closer to the actual situation by adding the stochastic failure algorithm and the crack-softening algorithm to the material. The test results in Reference [5] were used as the reference standard to verify the scientific and rationality of the numerical simulation method proposed in this paper by means of qualitative analysis and quantitative analysis.

## 2. Theoretical Foundations

### 2.1. Basic Theory of Mott Ring

**Figure 3:**
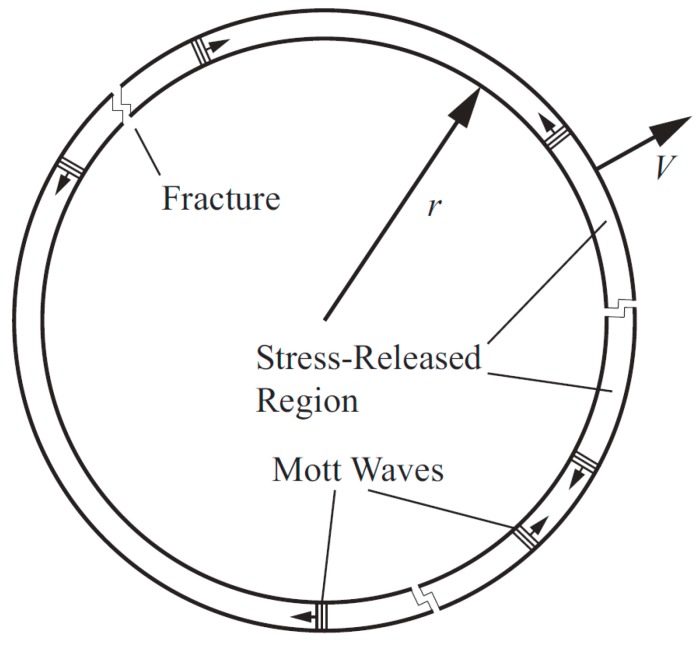
Schematic diagram of the Mott ring.

A Mott ring [15] is a one-dimensional structure with a uniform expansion. The material will break under the action of the circumferential tensile stress, and the location of generated crack is random, as shown in Figure 3. Once the cracks are generated, the unloading waves immediately propagate to both sides, resulting in stress unloading. The unloading wave is commonly known as a Mott wave. When the fracture is completed, the unloading area formed by the adjacent cracks becomes fragmented. In the Mott ring, the material is regarded as having rigid-ideal plasticity; that is, the wave front material is plastic and the post wave material is rigid. The tensile stress is the constant yield stress *Y*, and the strain rate $\dot{\varepsilon }=V/r$ is regarded as a constant.

**Figure 4:**
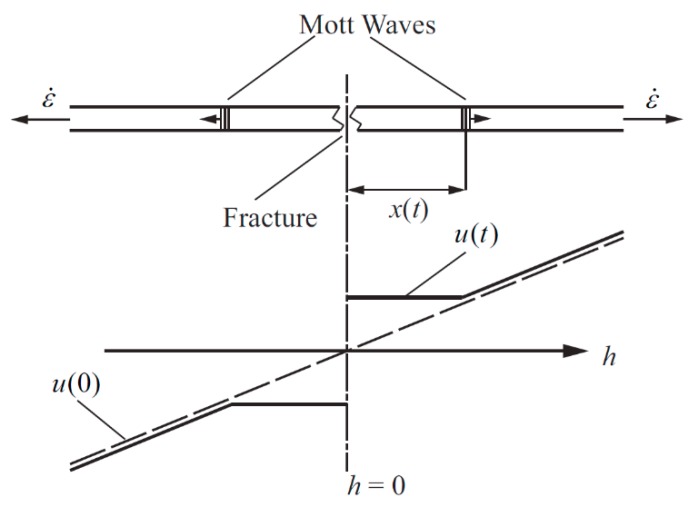
Circumferential velocity distribution of the Mott ring.

For the generation of a single crack in the Mott ring, Mott took a simplified approach. It is assumed that there is no unloading process in the material where the crack is generated, and the tensile stress is always zero. Therefore, the crack generation process and its energy loss can be ignored. Under the mechanical assumption of the Mott ring, the circumferential velocity distribution of the material is shown in Figure 4.

In Figure 4, the material position is *h*, the Mott wave position is *x*, and the circumferential velocity is *u*. The crack is generated at *t* = 0 and *h* = 0, and then the Mott waves propagate in both positive and negative directions. Since the material behind the wave is rigid, the circumferential velocity of the material is equal everywhere after the Mott wave, and the circumferential velocity *u*(*t*) can be expressed as:

(1) $$u\left(h,t\right)=\left\{ \begin{array}{ll} \dot{\varepsilon }x\left(t\right) & 0\leq h\leq x\left(t\right)\\ \dot{\varepsilon }h & x\left(t\right)\leq h\leq h_{0} \end{array}\right.$$

where *h*_0_ is the investigation distance. Within this distance, the total momentum can be expressed as follows:

(2) $$\rho \dot{\varepsilon }x^{2}+\int_{x}^{h_{0}}\rho \dot{\varepsilon }h\;dh=\frac{1}{2}\rho \dot{\varepsilon }\left(x^{2}+h_{0}^{2}\right)$$

Within the investigation distance, the change rate of total momentum over time is determined by the load. Since the material at the crack generation (*h* = 0) is not affected by the load, and the load at *h* = *h*_0_ is the constant yield stress *Y*, there is a momentum conservation relationship:

(3) $$\rho \dot{\varepsilon }x\frac{dx\;}{dt}=Y$$

By integrating Equation (3), the expression of a Mott wave position changing with time *x*(*t*) is obtained as follows:

(4) $$x\left(t\right)=\sqrt{\frac{2Yt}{\rho \dot{\varepsilon }}}$$

### 2.2. Grady One-Dimensional Fracture Theory and the Crack-Softening Algorithm

**Figure 5:**
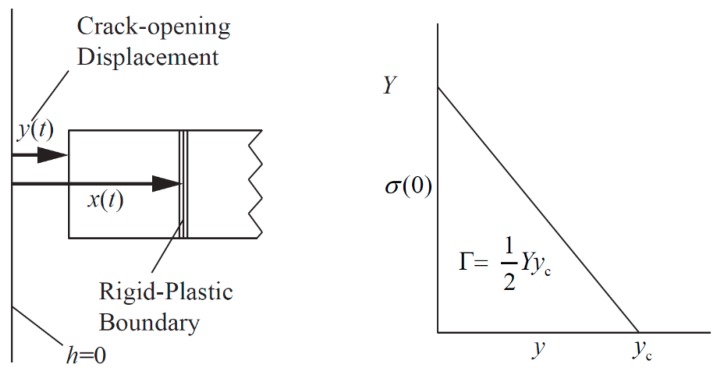
Schematic diagram of Grady one-dimensional fracture model.

Grady and Kipp [15,16,17] thought that the energy consumed by the crack generation cannot be ignored in some cases. Therefore, the following assumptions were proposed: the stress at the crack is not immediately unloaded, the tensile stress is gradually reduced from the yield stress *σ* = *Y* to *σ* = 0, and the unloading curve of the material is linear, as shown in Figure 5.

In Figure 5, *y*(*t*) is the crack-opening displacement, *σ*(0) is the tensile stress of the material where the crack occurs (*h* = 0), and Γ is the fracture energy. When *y*(*t*) reaches the critical value *y*_c_, the unloading process is considered complete. Since *σ*(0) is not zero, the momentum conservation relationship of a Mott ring can be rewritten as:

(5) $$\rho \dot{\varepsilon }x\frac{dx}{dt}=Y\frac{y}{y_{c}}=\frac{Y^{2}}{2\Gamma }y$$

In the Grady one-dimensional fracture model, the material still satisfies the rigid-ideal plasticity assumption, and there is a relationship: $dy/dt=\dot{\varepsilon }x$. Thus, the expression of the unloading length *x*(*t*) can be obtained as follows:

(6) $$x\left(t\right)=\frac{1}{12}\frac{Y^{2}}{\rho \Gamma }t^{2}$$

**Figure 6:**
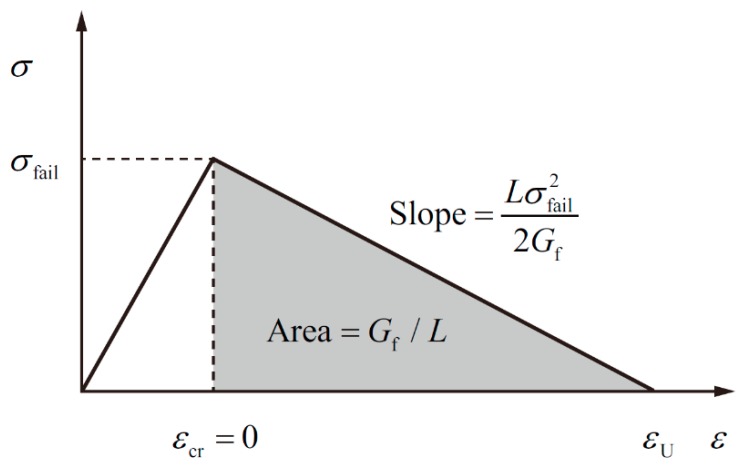
Schematic diagram of the crack-softening algorithm.

The crack-softening algorithm refers to the Grady one-dimensional fracture model, and its action process can be described as follows: when the grid reaches the tensile failure condition, it must undergo an unloading process to achieve complete failure; the grid stress–strain curve is linear, and the stress cannot exceed a certain critical value during the unloading process; when the grid is no longer subjected to the tensile stress, the unloading process will terminate. The action process of the crack-softening algorithm is shown in Figure 6.

In Figure 6, *σ*_fail_ represents the grid-failure stress; *ε*_cr_ represents the crack strain, which is the strain generated by the grid during the unloading process; *L* is the dimension along the grid stretching direction; and *G*_f_ is the fracture energy, corresponding to the above Γ. The unloading characteristics of the grids are determined by *G*_f_. When *ε*_cr_ reaches the critical value *ε*_U_, the unloading process is completed. Thus, Figure 6 can be expressed as an analytical expression:

(7) $$G_{f}=\int_{\varepsilon_{\mathrm{cr}}=0}^{\varepsilon_{\mathrm{cr}}=\varepsilon_{U}}\sigma Ld\varepsilon_{\mathrm{cr}}$$

The critical crack strain *ε*_U_ can be expressed as:

(8) $$\varepsilon_{U}=\frac{2G_{f}}{\sigma_{\mathrm{fail}}L}$$

During the unloading process, the maximum tensile stress *σ*_max_ that the grid can withstand is expressed as:

(9) $$\sigma_{\max}=\sigma_{\mathrm{fail}}\left(1-\mathrm{Dam}\right),\;\mathrm{Dam}=\frac{\varepsilon_{\mathrm{cr}}}{\varepsilon_{U}}$$

where Dam is the damage degree of the grid. Here, the following definitions are made:

Dam = 0: it represents the grid does not reach the failure condition.
Dam = 1: it represents the grid has gone through the unloading process and completely invalid.

In addition, when the grid is completely invalid, it will no longer be subjected to the tensile shear stress.

The main control parameter *G*_f_ of the crack-softening algorithm can be calculated from the value of *K*_f_ (dynamic fracture toughness) [18]. The relationship between the two parameters is:

(10) $$K_{f}=\sqrt{EG_{f}}$$

where *E* is the Young’s modulus of material, and *K*_f_ can be determined using the spallation test. For different materials, such as steels with different chemical compositions or heat treatment processes, the value of *K*_f_ usually differs greatly [16].

### 2.3. Grady Spallation Theory and Spall Strength of Materials

In the 1980s, Grady [19] conducted extensive research on the spall strength of condensed matter. He considered that when the condensed matter impacts the target at high speed, fracture will occur inside the material due to the tensile stress exceeding its tensile strength, and this phenomenon is called spallation. According to the microscopic mechanism of material fracture, the spallation is usually divided into brittle spallation and ductile spallation.

**Figure 7:**
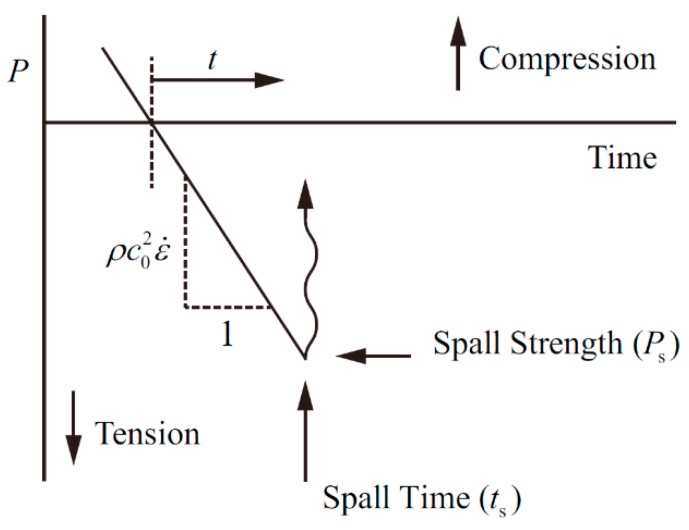
*P*-*t* relationship of the Grady spallation model.

The Grady spallation model considers that the material undergoes elastic volume deformation under linear tensile stress, and the strain rate is constant. The tensile stress of a material *P* can be expressed as:

(11) $$P=\rho c_{0}^{2}\dot{\varepsilon }t$$

where *ρ* is the material density, *c*_0_ is the material bulk velocity, $\dot{\varepsilon }$ is the volumetric strain rate, and *t* is the material tensile time. The above situation is illustrated in Figure 7.

**Figure 8:**
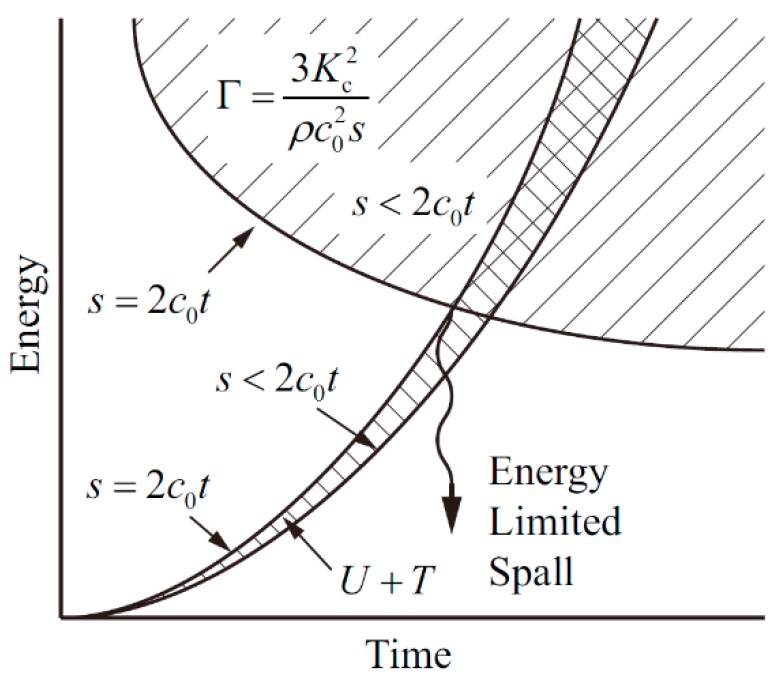
Schematic diagram of Grady brittle spallation model.

Under the action of tensile stress, the elastic volume and kinetic energy of a material gradually increases, and the material will crack when the total energy is greater than the energy required for crushing. The relationship between energy and time in the brittle spallation model is shown in Figure 8, and the energy condition for brittle spallation is as follows:

(12) $$\frac{1}{2}\frac{P^{2}}{\rho c_{0}^{2}}+\frac{1}{120}\rho {\dot{\varepsilon }}^{2}s^{2}\geq \frac{3K_{c}^{2}}{\rho c_{0}^{2}s}$$

where *P* is the tensile stress, *ρ* is the material density, *c*_0_ is the material bulk velocity, $\dot{\varepsilon }$ is the volumetric strain rate, and *s* is the fragment size. *K*_c_ is the static fracture toughness of material, which is close to the value of *K*_f_ (dynamic fracture toughness) mentioned above and can be interchanged [15,20].

The first term on the left side of Inequality (12) is the elastic volume energy *U*, the second term is the kinetic energy *T*, and the right side of Inequality (12) represents the energy required for brittle spallation *Γ*. Since there is the following relationship: *T* ≤ *U*/15, *T* can be ignored. In addition, the fragment size *s* must satisfy the following inequality:

(13) $$s\leq 2c_{0}t$$

Obviously, when the two Inequalities (12) and (13) take the equal sign at the same time, the energy required for spallation is the smallest, as shown in Figure 8. The spall strength *P*_s_ can be solved using the above two equations:

(14) $$P_{s}={\left(3\rho c_{0}K_{c}^{2}\dot{\varepsilon }\right)}^{1/3}$$

In the numerical simulation, the material has various tensile failure models, such as the hydrostatic pressure and principal stress failure models, taking a given hydrostatic pressure and principal stress as the material tensile failure criterion, respectively. Reference [21] shows that when the principal stress failure model is used, the tensile strength of a material can be estimated by the Grady spallation theory. In other words, when the actual tensile strength of a material is unknown, the principal tensile failure stress *σ*_T_ in the numerical simulation can be replaced by the theoretical spall strength *P*_s_. Therefore, in the following numerical simulation, the principal stress failure model will be added to the metal core material of a PELE projectile.

### 2.4. Mott Fracture Probability Density Function and Stochastic Failure Algorithm

In order to solve the problem of multiple cracks, Mott proposed to use the fracture probability density function *λ*(*ε*) to control the occurrence probability of cracks, and these functions are called the risk function and the conditional failure function. The expression *λ*(*ε*)d*ε*d*l* is the probability that a crack will occur in the element with a length d*l* and strain *ε* when the strain increases by d*ε*. In 1943, Mott [15] proposed three kinds of fracture probability functions *λ*(*ε*), which are:

(15) $$\lambda \left(\varepsilon \right)=\lambda_{0},\;(\mathrm{constant})$$

(16) $$\lambda \left(\varepsilon \right)=\frac{n}{\sigma }\;{\left(\frac{\varepsilon }{\sigma }\right)}^{n-1},\;n\geq 1$$

(17) $$\lambda \left(\varepsilon \right)=Ce^{\gamma \varepsilon }$$

where *n* and *σ* are the Weibull distribution constants, and *C* and *γ* are the Gumbel distribution constants. In the above three forms of functions, the exponential expression is the most commonly used.

It is assumed that the sample is composed of *N*_0_ unit length segments, each segment is stretched independently at the same strain rate, and the number of surviving segments is *N*. As a result, there is the following relationship:

(18) $$\frac{dN}{N}=-\lambda \left(\varepsilon \right)d\varepsilon$$

The above formula can also be rewritten as an expression in the integral form:

(19) $$N=N_{0}e^{-\int \lambda (\varepsilon )\;d\varepsilon }$$

Hence, the cumulative probability distribution of the whole sample before or at the strain *ε* can be obtained:

(20) $$F\left(\varepsilon \right)=1-\frac{N}{N_{0}}=1-e^{-\int \lambda \left(\varepsilon \right)d\varepsilon }$$

By differentiating the above formula, the complementary cumulative probability density can also be obtained:

(21) $$f\left(\varepsilon \right)=\frac{dF\left(\varepsilon \right)}{d\varepsilon }=\lambda \left(\varepsilon \right)e^{-\int \lambda \left(\varepsilon \right)d\varepsilon }$$

The fracture frequency function *λ*(*ε*) is different for different materials, resulting in different expressions for the cumulative probability distribution function *F*(*ε*) and the cumulative probability density function *f*(*ε*).

The stochastic failure algorithm refers to the Mott stochastic failure theory, which discretizes the Mott theory. Each grid is automatically assigned a stochastic coefficient, and the size of the coefficient is the ratio of the true failure strain of grid to the rupture strain of the material given by the user. The relationship between the grid number and the stochastic factor is determined by the given stochastic constant *γ*.

In the AUTODYN simulation software, the stochastic failure algorithm is embedded in order to more realistically describe the fracture and failure of a material [18]. The expression of cumulative failure probability is:

(22) $$P\left(\varepsilon_{N}\right)=1-\exp\left[-\frac{C}{\gamma }\left(\exp\left(\gamma \varepsilon_{N}\right)-1\right)\right]$$

where $\varepsilon_{N}$ is the normalized strain, i.e., $\varepsilon_{N}=\varepsilon /\varepsilon_{c}$; $\varepsilon_{c}$ is the fracture strain; and *γ* and *C* are the material constants. For Equation (22), there is a very important premise condition: when the material strain reaches the specified fracture strain, the failure probability of the grid is 50%. That is, there exists the following relationship:

(23) $$P\left(\varepsilon_{N}=1\right)=0.5$$

Thus, the relationship between *γ* and *C* can be obtained:

(24) $$C=\frac{\gamma \ln2\;}{e^{\gamma }-1}$$

Substituting Equation (24) into Equation (22), we can get:

(25) $$P\left(\varepsilon_{N}\right)=1-\exp\left[K\left(\exp\left(\gamma \varepsilon_{N}\right)-1\right)\right],\;K=\frac{\ln2}{1-e^{\gamma }}$$

Similarly, by differentiating the above formula, the cumulative failure probability density expression can be obtained:

(26) $$f\left(\varepsilon_{N}\right)=-K\gamma \exp\left[\left(\gamma \varepsilon_{N}\right)+K\left(\exp\left(\gamma \varepsilon_{N}\right)-1\right)\right]$$

**Figure 9:**
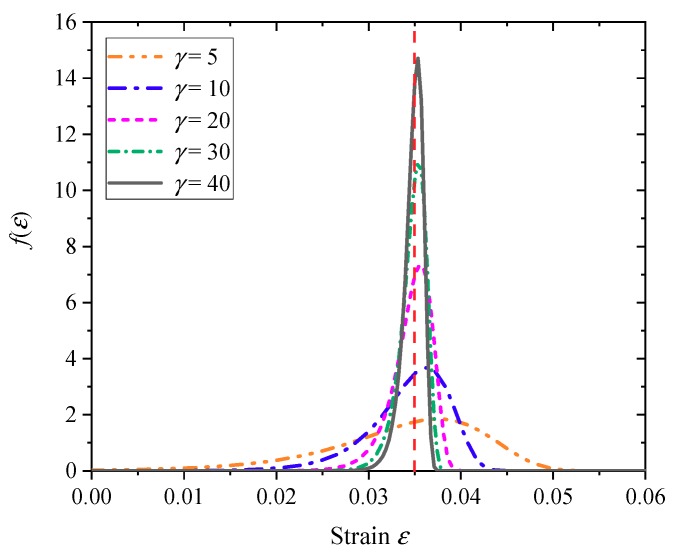
Cumulative failure probability density distribution function.

**Figure 10:**
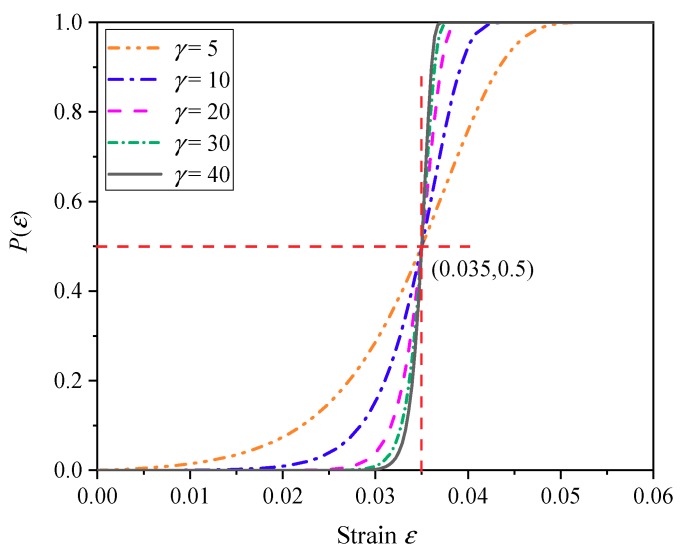
Cumulative failure probability distribution function.

Since the *γ*–*C* relationship is certain, it is only necessary to give a specific *γ* value to determine the stochastic dispersion of grids in actual operation. However, for materials with unknown fracture properties, the *γ* value can only be selected empirically. Under the condition that the given fracture strain is $\varepsilon_{c}=0.035$, the cumulative failure probability density *f*(*ε*) and the cumulative fracture probability *P*(*ε*) under different *γ* values are shown in Figure 9 and Figure 10, respectively.

As can be seen from Figure 9, the material fracture probability density curve *f*(*ε*) becomes steeper as the *γ* value increases. The failure strain error is defined as the error between the strain at the center of the fracture probability density distribution *f*(*ε*) and the given fracture strain $\varepsilon_{c}$, and the failure strain error decreases as the *γ* value increases. From this point of view, it is desirable that the *γ* value should be as large as possible. However, it can also be seen from Figure 10 that if the *γ* value is too large, the failure strain of the grid will become more concentrated, that is to say, the material crushing performance will tend to be uniform. In this respect, it is not desirable that the *γ* value is too large. Therefore, for a specific material, there is an optimal range of the *γ* value. In the following numerical simulation, the optimal *γ* value will be determined by trial calculation.

## 3. Numerical Simulation

In this paper, the nonlinear dynamics software AUTODYN (Century Dynamics, Fort Worth, TX, USA) [22] was used to simulate the crushing process of PELE casing material. AUTODYN is an explicit finite element analysis program, which is used to solve the highly nonlinear dynamic problems of solids, fluids, gases, and their interactions. More importantly, the software has a unique stochastic failure model, which can reflect the randomness and non-uniformity exhibited by the material.

### 3.1. Finite Element Model

**Figure 11:**
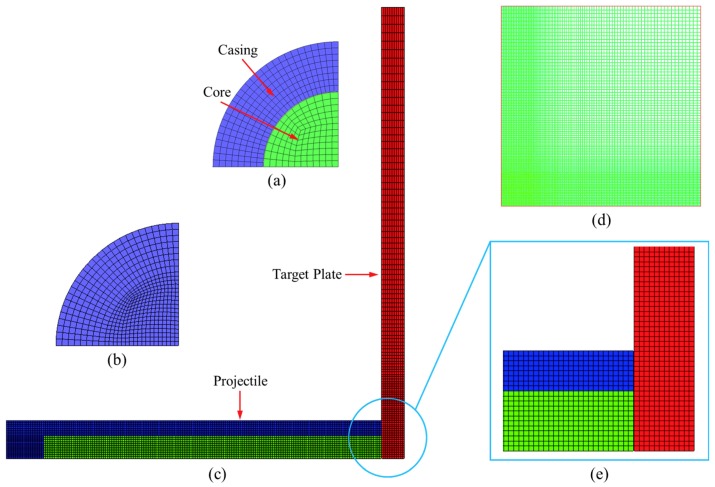
Schematic diagram of the finite element model. (**a**) Projectile head, (**b**) Projectile rear, (**c**) FE model, (**d**) Target plate grid, and (**e**) Impact region.

The entire model was divided into three parts: the outer casing of the PELE projectile, the inner core of the PELE projectile, and the target plate. The PELE projectile length was 50 mm, the outer and inner diameters of the projectile were 10 mm and 6 mm, respectively, and the thickness of the projectile rear was 5 mm. The inner core was a ø6 mm× 45 mm cylinder. The length and width of the target plate were both 120 mm, and the thickness was selected according to different working conditions. The unstructured hexahedral mesh was used in both the projectile and target plate, and the average size of the grid was 0.25 mm. The whole model was established using HyperMesh software (Altair Engineering, Detroit, MI, USA), then the finite element model was imported into AUTODYN for the solution and the Lagrange algorithm was adopted. In order to reduce the number of grids and improve the computational efficiency, the meshing method of the target plate adopted the variable-step size method. The grids were denser in the center area, which was about two times the diameter of projectile, and the minimum grid size was 0.25 mm. The transmissive boundary condition was applied to the edge of the target plate. In the case of considering the vertical impact, the model adopted a 1/4 simplification; when considering the oblique penetration, the model adopted a 1/2 simplification; when considering the projectile rotation, the model was not simplified. The schematic diagram of the finite element model is shown in Figure 11.

### 3.2. Determination of Key Parameters in the Material Failure Model

In this paper, the experiments carried out by Paulus [5] were used as the reference for comparison. In the reference experiments, the projectile casing was made of tungsten alloy (D-180 K), the inner core was made of aluminum (A-G3) and polyethylene (PE), and the target plate material was made of steel (XC48) and aluminum (A-U4G), but several experimental materials were not identical in the material library of AUTODYN. In order to solve this key problem, the materials were of the same type as the experimental materials, but different parameters were found in the material library of AUTODYN, which have the same equation of state and constitutive equation as the experimental materials. Then, all material parameters in the substitute material selected in AUTODYN were replaced with the parameters of the experimental material, which could ensure the consistency of the materials in the experiment and numerical simulation.

**Figure 12:**
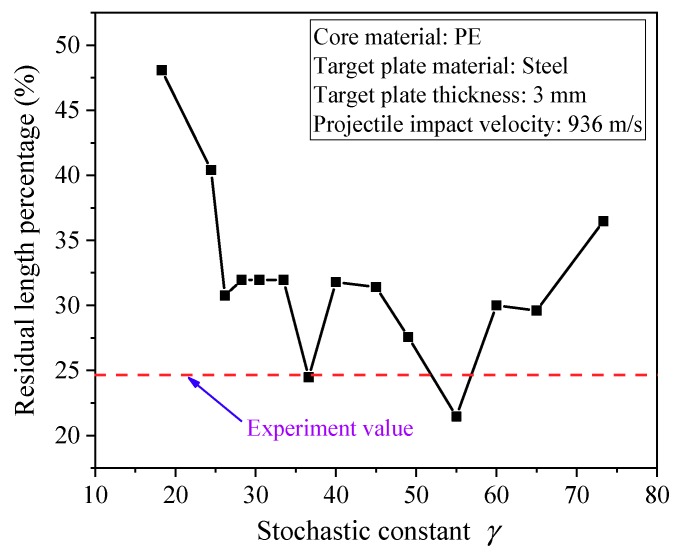
Trial results under different *γ* values.

(1) Determination of the key parameter *G*_f_ of the crack-softening algorithm of the outer casing tungsten: The fracture energy *G*_f_ was determined based on the dynamic fracture toughness *K*_f_ and the Young’s modulus *E*. Reference [23] provided the range of the dynamic fracture toughness of the fragile tungsten alloy *K*_f_ = 3–5 MPa⋅m^1/2^, and this paper takes the intermediate value *K*_f_ = 4 MPa⋅m^1/2^. Then, according to *G*_f_ = *K*_f_/*E* (see Equation (10)) and *E* = 360 GPa, *G*_f_ = 45 J/m^2^ was obtained.

(2) Determination of the key parameter *γ* of the stochastic failure algorithm of the outer casing tungsten: The stochastic constant *γ* was determined by the failure strain error analysis and numerical trial calculations. The test condition (the inner core material was polyethylene, the target plate was 3 mm thick steel, and the projectile velocity was 936 m/s) in Reference [5] was used as the reference standards. A series of *γ* values were substituted into the numerical simulation, and it was found that the length of the residual projectile varied greatly when the *γ* value was different, and the trial result is shown in Figure 12.

As shown in Figure 12, with the increase of the *γ* value, the changing law of the residual projectile length was rather complicated, and the simulation results at *γ* = 36.5 were in good agreement with the experimental results. Therefore, the stochastic constant in this paper was identified as *γ* = 36.5.

(3) Determination of the key parameter of principal stress failure model of the inner core material Al-6061: The tensile strength *σ*_T_, corresponding to *P*_s_ above, could be calculated by the Grady spallation theory, where its specific expression is: $\sigma_{T}={\left(3\rho c_{0}K_{f}^{2}\dot{\varepsilon }\right)}^{1/3}$. According to Reference [15], the dynamic fracture toughness was taken as *K*_f_ = 27.5 MPa⋅m^1/2^, and the strain rate was taken as $\dot{\varepsilon }$ ≈ 10^3^−10^4^ s^−1^. Then, when the material density *ρ*_0_ = 2.65 g/cm^3^ and the volumetric sound velocity *c*_0_ = 5176 m/s were substituted into the expression, we obtained *σ*_T_ ≈ 0.31–0.68 GPa. Here, we take the intermediate value σ_T_ = 0.5 GPa.

### 3.3. Material Model and Parameters

In this paper, the selection method of the equation of state and constitutive equations for all materials are referred to References [24,25]. The equation of state for all materials adopted Mie-Grüneisen, which is denoted as the “shock” equation of state [22] in AUTODYN, and its basic theoretical relationship is as follows:

(27) $$U_{s}=c_{0}+s\cdot u$$

where *U*_s_ is the shock wave velocity, *u* is the post-wave particle velocity, and *c*_0_ and *s* are the material Hugoniot parameters.

The constitutive equation of the material is elastic-ideal plastic. The constitutive law is based on the von Mises yield criterion and the steady yield stress assumption, and the shear modulus of the material is regarded as a constant. This constitutive equation is described as the “von Mises” strength model in AUTODYN, which requires a given material shear modulus *G* and the flow stress *Y*. In addition, the dynamic yield strength is typically approximately 2–3 times the static value.

The projectile casing adopted the principal stress/strain failure model. It was considered that the material would fail when the tensile principal strain reached *ε*_T_ or the tensile principal stress reached *σ*_T_. According to Reference [5], *ε*_T_ = 0.035 and *σ*_T_ = 2.8 GPa. In addition, the stochastic failure algorithm and crack-softening algorithm were added to the projectile casing material. According to the analysis above, the stochastic coefficients were *γ* = 36.5 and *G*_f_ = 45 J/m^2^. The aluminum core material adopted the principal stress failure model, and the tensile strength was *σ*_T_ = 0.5 GPa, the polyethylene core material was not added with failure. The material of the target plate adopted the plastic strain failure model, and it was considered that the material would fail when the plastic strain reached a certain value. All materials were added with an artificial erosion algorithm (Erosion) to ensure a normal calculation. The target plate material adopted the failure erosion algorithm (Failure), which was used to delete the invalid grid. The rest of the materials use the geometric strain erosion algorithm, which removed the grid whose instantaneous geometric strain (Geometric Strain) was greater than the given value. In summary, the material parameters used in this paper are summarized in Table 1.

## 4. Analysis and Discussion of the Simulation Results

### 4.1. Influence of the Crack-Softening Algorithm and Stochastic Failure Algorithm on the Simulation Results

**Figure 13:**
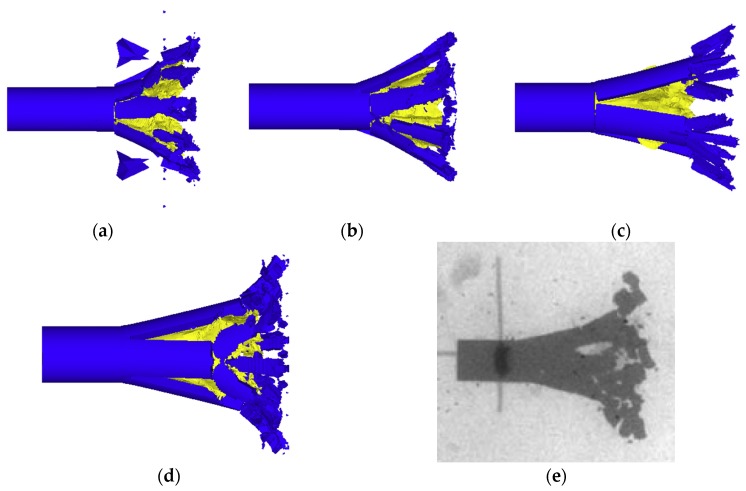
Comparison of the test results and simulation results of different failure models: (**a**) no crack softening, (**b**) no stochastic failure, (**c**) no crack softening and no stochastic failure, (**d**) crack softening and stochastic failure, and (**e**) X-ray experiment picture.

In order to more intuitively give the influence of the crack-softening algorithm and stochastic-failure algorithm on the simulation results, four groups of simulation conditions ((a) no crack softening, (b) no stochastic failure, (c) no crack softening and no stochastic failure, and (d) crack softening and stochastic failure) were designed in this paper. Moreover, the test conditions (the inner core material was polyethylene, the target plate was 8 mm thick aluminum, and the projectile velocity was 939 m/s) in Reference [5] were taken as the reference. The simulation results corresponding to each group of conditions were compared with the experimental results. The effects of adding the crack-softening algorithm and stochastic-failure algorithm before and after are shown in Figure 13.

As shown in Figure 13, when the crack-softening algorithm (a, c) was not added, the material failure was not in accordance with the actual situation. Moreover, the failure grids were usually distorted so that more grids were eroded, and the remaining failure area of material was usually smaller. When the stochastic failure algorithm was not added (b, c), the fragment length of projectile was larger and the shape of fragments was not ideal. After adding the stochastic-failure algorithm and crack-softening algorithm (d), the simulation results were more consistent with the actual conditions (e). The length and shape of fragments were ideal, and the projectile failure region was relatively uniform.

In summary, adding the crack-softening algorithm was beneficial to control the distortion of the failure grids, and adding the stochastic failure algorithm was beneficial to control the length and shape of fragments. Therefore, the crack-softening algorithm and stochastic-failure algorithm were conducive to simulating the real damage situation, and they were added in the subsequent simulation.

### 4.2. Quantitative Comparison of the Simulation Results and Experimental Results

Based on the analysis in Section 4.1, it can be qualitatively seen that the simulation results after adding the two failure algorithms were in good agreement with the experimental results. However, this chapter hopes to quantitatively analyze the coincidence degree between the simulation results and the experimental results by designing several sets of simulation conditions. The entire qualitative comparison process was based on two parameters: the residual velocity of the projectile and the maximum radial velocity of fragments. Referring to the experimental results in Reference [5], the specific setting of the simulation conditions in this paper were designed as shown in Table 2.

**Figure 14:**
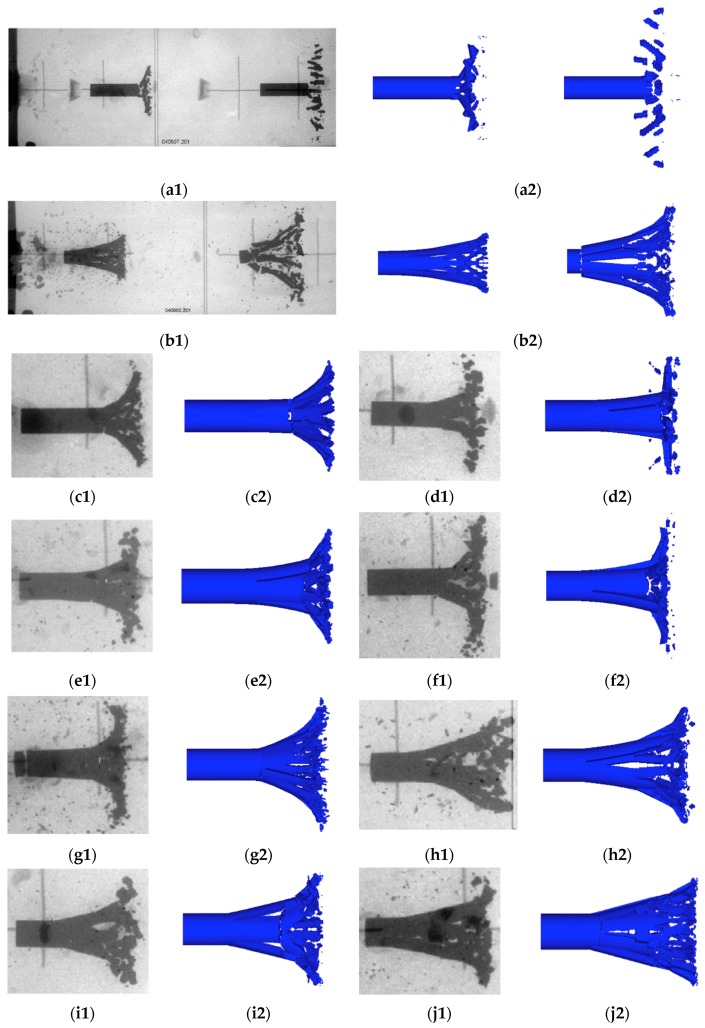
Comparison of test results and simulation results under different conditions: (**a1**) #1 experiment result; (**a2**) #1 simulation result; (**b1**) #8 experiment result; (**b2**) #8 simulation result; (**c1**) #2 experiment result; (**c2**) #2 simulation result; (**d1**) #3 experiment result; (**d2**) #3 simulation result; (**e1**) #4 experiment result; (**e2**) #4 simulation result; (**f1**) #5 experiment result; (**f2**) #5 simulation result; (**g1**) #6 experiment result; (**g2**) #6 simulation result; (**h1**) #7 experiment result; (**h2**) #7 simulation result; (**i1**) #9 experiment result; (**i2**) #9 simulation result; (**j1**) #10 experiment result; (**j2**) #10 simulation result.

The numerical simulation results were compared with the flash X-ray pictures [5] as shown in Figure 14. The maximum radial velocity of fragments and the residual velocity of the projectile obtained by the simulation are shown in Table 3.

It can be seen intuitively from Figure 14 and Table 3 that the simulation results were basically consistent with the experimental results and the errors were smaller. In addition, it was also found that the PELE projectile crushing performance was also greatly different due to the inner core material, and target plate material and thickness, which was embodied in the following aspects:

(1) Comparing the following four sets of working conditions (#3–#9; #4–#10; #5–#7; #6–#8), the following conclusions can be obtained intuitively from Figure 14 and Table 3. When the core material was aluminum, the residual length of the projectile was larger, the size of fragments was smaller, and the radial velocity of fragments was higher than when the core material was polyethylene.
(2) Comparing the following two sets of working conditions (#1–#5; #2–#6) from Table 3, for the same core material and the target plate thickness, when the target material changed from aluminum to steel, the residual velocity of projectile decreases and the radial velocity of fragments increased. Comparing the following two sets of working conditions (#1–#3; #2–#4) from Table 3, for the same core material and the target plate material, when the target plate thickness increased, the residual velocity of the projectile decreased and the radial velocity of the fragments increased.

**Figure 15:**
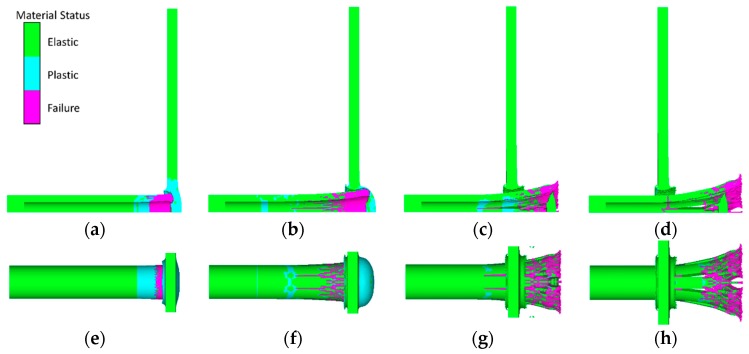
Fragmentation process of a PELE projectile impacting the target plate: (**a**) *t* = 2 μs side view, (**b**) *t* = 7 μs side view, (**c**) *t* = 16 μs side view, (**d**) *t* = 2 μs side view, (**e**) *t* = 2 μs over view, (**f**) *t* = 7 μs over view, (**g**) *t* = 16 μs over view, and (**h**) *t* = 24 μs over view.

Taking the #6 simulation condition as an example, the fragmentation process of the PELE projectile impacting the target plate is shown in Figure 15. In order to facilitate the observation of the crushing process of the outer casing, the core material is not shown. The core material was aluminum, the target plate was 3 mm steel, and the projectile impact velocity was 1262 m/s.

As shown in Figure 15, at the moment of impacting the target plate, two shock waves propagating respectively toward the rear of the projectile and the back of the target plate were generated at the interface, and the plastic deformation of the projectile head caused the failure of the grids (a, e). While the plastic deformation region propagated toward the rear of projectile, the failure region of casing gradually expanded backward (b, f) under the combined action of the resistance of the target plate and the radial force of the inner core, and these failure regions were considered to be cracks. After the projectile perforated the target plate, the plug and the inner core continued to interact, and the casing cracks expanded further along the axial direction (c, g). When the projectile moved further forward, the fragments generated by the crack propagation gradually scattered under the radial velocity (d, h).

**Figure 16:**
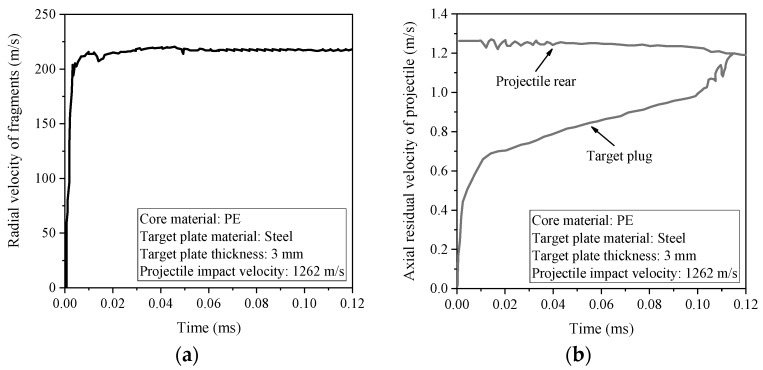
(**a**) Radial velocity time history curve of the material at the Gauss point of projectile casing, and (**b**) axial velocity time history curves of the material at the Gauss point of projectile casing and the target plug.

In the numerical simulation, a series of Gauss observation points were set up on the outer casing and the target plug to record the velocity changes. The radial velocity–time history curve of the material at the Gauss point of projectile casing is shown in Figure 16a. The axial velocity–time history curves of the material at the Gauss point of the projectile casing and target plug are shown in Figure 16b.

It can be seen from Figure 16a that the radial velocity of the fragments increased rapidly at ≈0–0.01 ms, because there existed a radial compression during this period. Then, some fluctuations occurred and it reached stability at about 0.05 ms, which indicates that the projectile had perforated the target plate. According to Figure 16b, the axial velocity of the projectile was gradually reduced under the resistance of the target plate. However, the axial velocity of the target plug was gradually increased under the interaction of the projectile and inner core. Finally, the two curves tend to be consistent around 0.115 ms, at which point the projectile and the target plug were no longer interacting. In addition, it was also found that the aluminum core projectile and the target plug have a shorter action time than the polyethylene core projectile.

## 5. Conclusions

The crushing mechanism of the outer casing of the PELE projectile during the penetration process was similar to the crushing theory of the Mott ring. This paper first introduced the theoretical work of Mott and Grady on the Mott ring problem and the Grady spallation theory briefly. According to the theoretical analysis, the material failure model parameters (stochastic constant *γ*, fracture energy *G*_f_, and tensile strength *σ*_T_) of the stochastic failure algorithm and the crack softening algorithm that were used in the numerical simulation were determined. Based on the obtained numerical simulation results, the following conclusions could be drawn:

(1) Based on the qualitative analysis designed in this paper, the crack-softening algorithm was beneficial for controlling the distortion of failure grids, and the stochastic-failure algorithm was beneficial to control the length and shape of fragments. After adding the two algorithms, the simulation results were more consistent with the actual situation.
(2) Several sets of simulation conditions were designed to quantitatively analyze the coincidence degree between the simulation and experiment by means of the residual velocity of the projectile and the maximum radial velocity of fragments. The results indicated that the simulation results were in good agreement with the experimental results and the errors were small. Specifically, the radial velocity error of fragments was within 8.5%, and the axial residual velocity error of the projectile was within 2%.
(3) According to the four sets of working conditions (#3–#9; #4–#10; #5–#7; #6–#8) shown in Figure 14 and Table 3, the following conclusion could be obtained intuitively: when the inner core material was aluminum, the residual length of projectile was larger, the size of fragments was smaller, and the radial velocity of fragments was higher than the situation where the inner core material was polyethylene.
(4) For the same core material and target plate thickness, when the target material changed from aluminum to steel, the residual velocity of the projectile decreased and the radial velocity of the fragments increased, which could be explained intuitively by the two groups of working conditions (#1–#5; #2–#6) in Table 3. Similarly, for the same core material and target plate material, when the target plate thickness increased, the residual velocity of the projectile decreased and the radial velocity of the fragments increased, which could also be explained intuitively by the two groups of working conditions (#1–#3; #2–#4) in Table 3.

In summary, the research ideas and conclusions are feasible and scientific. The numerical simulation method in this paper can provide reference for rapidly and accurately analyzing the influencing factors of PELE projectiles, which has important engineering application value.

## Figures and Tables

**Table 1:** Material parameters in the numerical simulation.

Variable	Material				
	Tungsten	Al-6061	PE	Al-7075	Steel-4340
*ρ*_0_ (g/cm^3^)	18	2.65	0.92	2.8	7.823
*c*_0_ (km/s)	4.03	5.24	2.9	5.2	4.57
*s*	1.237	1.4	1.48	1.36	1.49
Grüneisen Coefficient	-	1.97	1.6	-	-
*C*_p_ (J/kg∙K)	-	885	2300	-	-
Shear Modulus *G* (GPa)	139.02	27.5	0.13	26.7	77
Yield Stress *Y* (GPa)	1.5	0.3	0.02	0.4	0.8
Principle Tensile Stress *σ*_T_ (GPa)	2.8	0.5	-	-	-
Principle Tensile Strain *ε*_T_	0.035	-	-	-	-
Fracture Energy *G*_f_ (J/m^2^)	45	-	-	-	-
Stochastic Variance *γ*	36.5	-	-	-	-
Inst. Geometric Strain	0.6	0.8	1.8	Failure	Failure

**Table 2:** Simulation conditions for quantitative analysis.

Condition Number	Inner Core Material	Target Plate Material	Target Plate Thickness (mm)	Projectile Impact Velocity (m/s)
#1	Al	Al	3	929
#2	Al	Al	3	1275
#3	Al	Al	8	937
#4	Al	Al	8	1254
#5	Al	Steel	3	925
#6	Al	Steel	3	1261
#7	PE	Steel	3	936
#8	PE	Steel	3	1262
#9	PE	Al	8	939
#10	PE	Al	8	1258

**Table 3:** Comparison of the maximum radial velocity of fragments and the axial residual velocity of the projectile between the simulation results and experimental results.

Condition Number	Maximum Radial Velocity of Fragments (m/s)		Error	Axial Residual Velocity of Projectile (m/s)		Error
	Experiment	Simulation		Experiment	Simulation	
#1	112	114	1.8%	914	921	0.7%
#2	158	149	−5.7%	1261	1259	−0.2%
#3	143	139	−2.7%	900	907	0.8%
#4	221	207	−6.3%	1208	1219	0.9%
#5	184	192	4.3%	895	904	1.0%
#6	243	229	−5.8%	1231	1246	1.2%
#7	94	102	8.5%	889	907	2.0%
#8	145	134	−7.5%	1206	1221	1.2%
#9	112	116	3.6%	887	904	1.9%
#10	184	171	−7.1%	1203	1224	1.7%

## References

[1] Kesberg G., Schirm V., Kerk S. PELE: The future ammunition concept. Proceedings of the 21st International Symposium on Ballistics (ISB’21).

[2] Paulus G., Chanteret P.Y., Wollmann E. PELE: A new penetrator concept for generating lateral effects. Proceedings of the 21st International Symposium on Ballistics (ISB’21).

[3] Rheinmetall Waffe Munition 105/120/125 mm PELE Firing Results. https://ndiastorage.blob.core.usgovcloudapi.net/ndia/2005/garm/wednesday/borngen.pdf.

[4] Gloude D. Capabilities of Penetrator with Enhanced Lateral Efficiency. https://ndiastorage.blob.core.usgovcloudapi.net/ndia/2007/gun_missile/GMTueAM1/GloudePresentation.pdf.

[5] Paulus G., Schirm V. (2006). Impact behavior of PELE projectiles perforating thin target plates. Int. J. Impact Eng..

[6] Zhu J., Zhao G., Du Z., Wang X. (2009). Mechanism of PELE projectiles perpendicularly impacting on thin target plates. Explos. Shock Waves.

[7] Zhu J., Zhao G., Du Z., Li D. (2007). Experimental study of the influence factors on small caliber PELE impacting thin target. Chin. J. Exp. Mech..

[8] Jiang J., Zhang M., Men J., Wang S. (2010). Experimental study on multi-layered target penetration of PELE with different cores. Trans. Beijing Inst. Technol..

[9] Tu S., Wang J., An Z., Chang Y. Influence of thickness of armor on the burst-effect of steel shell PELE. Proceedings of the 9th International Conference on Electronic Measurement & Instruments.

[10] Du Z., Song L. (2011). Theoretical model of penetrator with enhanced lateral effect impacting thin metal target. J. Nanjing Univ. Sci. Technol..

[11] Fan Z., Ran X., Tang W., Ke Y., Li Z. (2016). The model to calculate the radial velocities of fragments after PELE penetrator perforating a thin plate. Int. J. Impact Eng..

[12] Verreault J., Hinsberg V.N., Abadjieva E. PELE fragmentation dynamics. Proceedings of the 27th International Symposium on Ballistics (ISB’27).

[13] Wang H., Ji P., Yu Q., Zheng Y. (2010). Numerical simulation of oblique penetration of PELE into finite thickness plates. Trans. Beijing Inst. Technol..

[14] Verreault J. (2015). Analytical and numerical description of the PELE fragmentation upon impact with thin target plates. Int. J. Impact Eng..

[15] Grady D. (2006). Fragmentation of Rings and Shells: The Legacy of NF Mott.

[16] Grady D., Kipp M. (1997). Fragmentation properties of metals. Int. J. Impact Eng..

[17] Kipp M.E., Grady D. (1985). Dynamic fracture growth and interaction in one dimension. J. Mech. Phys. Solids.

[18] Century Dynamics Inc. (2003). AUTODYN Theory Manual.

[19] Grady D. (1988). The spall strength of condensed matter. J. Mech. Phys. Solids.

[20] Grady D., Olsen M. (2003). A statistics and energy based theory of dynamic fragmentation. Int. J. Impact Eng..

[21] Livingstone I., Verolme K., Hayhurst C. (2001). Predicting the fragmentation onset velocity for different metallic projectiles using numerical simulations. Int. J. Impact Eng..

[22] Century Dynamics Inc. (2003). Interactive Non-Linear Dynamic Analysis Software AUTODYN User Manual.

[23] Grady D. (1999). Impact failure and fragmentation properties of tungsten carbide. Int. J. Impact Eng..

[24] Rosenberg Z., Dekel E. (2009). On the deep penetration and plate perforation by rigid projectiles. Int. J. Solids Struct..

[25] Rosenberg Z., Dekel E. (2010). On the deep penetration of deforming long rods. Int. J. Solids Struct..

